# Osteoclast visualization: Tartrate-resistant acid phosphatase activity staining using NewFuchsin compatible with non-aqueous mounting and tissue clearing

**DOI:** 10.1016/j.mex.2024.103136

**Published:** 2024-12-26

**Authors:** Takashi Nakamura, Katsuhiro Kawaai, Yukiko Kuroda, Koichi Matsuo

**Affiliations:** a Department of Biochemistry, Tokyo Dental College, 2-9-18 Kanda-Misakicho, Chiyoda-ku, Tokyo 101-0061, Japan; b Oral Health Science Center, Tokyo Dental College, 2-9-18 Kanda-Misakicho, Chiyoda-ku, Tokyo 101-0061, Japan; c Laboratory of Cell and Tissue Biology, Keio University School of Medicine, 35 Shinanomachi, Shinjuku-ku, Tokyo 160-8582, Japan

**Keywords:** Osteoclast staining, Bone section, Dehydration, Non-aqueous mounting, Bone, TRAP, Acp5, Organic solvent, Mounting media, Tissue clearing, NewFuchsin TRAP staining

## Abstract

Tartrate-resistant acid phosphatase (TRAP) staining is widely used to stain osteoclasts in histological bone sections. The red dye formed by the conventional TRAP enzymatic reaction using naphthol AS-MX (or AS-BI) phosphate and fast red-violet (or garnet) chromogens is readily soluble in alcohol or xylene and requires air-drying prior to cover slipping or the use of an aqueous mounting medium. However, the use of an aqueous mounting medium makes it difficult to store stained specimens for a long time. In this modified method, a new fuchsin (NewFuchsin) was used as a chromogen, which enabled dehydration and clearing after staining and the use of a non-aqueous organic solvent-based mounting medium. Samples prepared using this modified TRAP activity staining method (NewFuchsin TRAP staining) have the following advantages over conventional TRAP staining:

• The staining of sections provides a clear histological image and allows for long-term preservation.
• The red dye formed by NewFuchsin TRAP staining can be detected not only in the bright field, but also in the fluorescent field.
• Combined with tissue clearing using ethyl cinnamate, osteoclasts are observed using three-dimensional imaging.

## Graphical abstract

**Figure:**
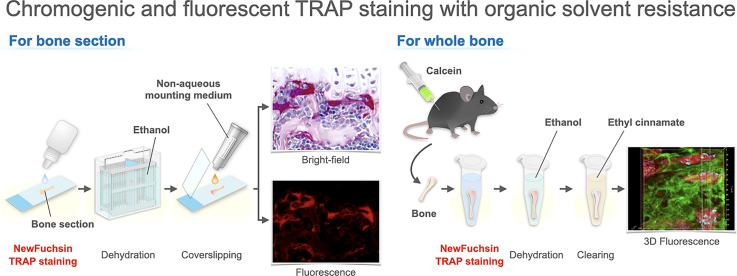

Specifications table

**Table:** 

Subject area:	Medicine and Dentistry
More specific subject area:	Bone histology
Name of your method:	NewFuchsin TRAP staining
Name and reference of original method:	TRAP staining A. A. Cole and L. M. Walters, Tartrate-resistant acid phosphatase in bone and cartilage following decalcification and cold-embedding in plastic, J. Histochem Cytochem. 35 (1987) 203–206.
Resource availability:	Not applicable

## Background

Osteoclasts are multinucleated cells that differentiate from hematopoietic cells of the monocyte-macrophage lineage. Osteoclasts resorb bone by attaching to the bone surface, forming a sealing zone, and releasing protons and proteases, such as cathepsin K, toward the bone surface. Since osteoclasts produce high levels of tartrate-resistant acid phosphatase (TRAP), TRAP staining is widely used to detect osteoclasts based on its enzymatic activity [1]. However, in conventional TRAP staining using fast red violet LB or fast garnet GBC chromogen and either naphthol AS-MX phosphate or naphthol AS-BI phosphate, the red dye produced is soluble in organic solvents and cannot be dehydrated with ethanol or cleared with xylene. Therefore, an organic solvent-based mounting medium is incompatible with conventional TRAP staining; instead, glycerol or aqueous mounting media are used. However, glycerol and aqueous mounting media are susceptible to bubble formation. Furthermore, these media impede long-term storage and diminish the image resolution, which are common problems faced by bone researchers. Fluorescent TRAP staining using an ELF97 substrate has also recently been reported; however, as with the above method, long-term preservation is difficult [2,3]. In this method article, we propose a TRAP activity staining technique suitable for long-term preservation and three-dimensional (3D) imaging.

## Method details

### NewFuchsin TRAP staining for decalcified bone section

**Fig. 1:**
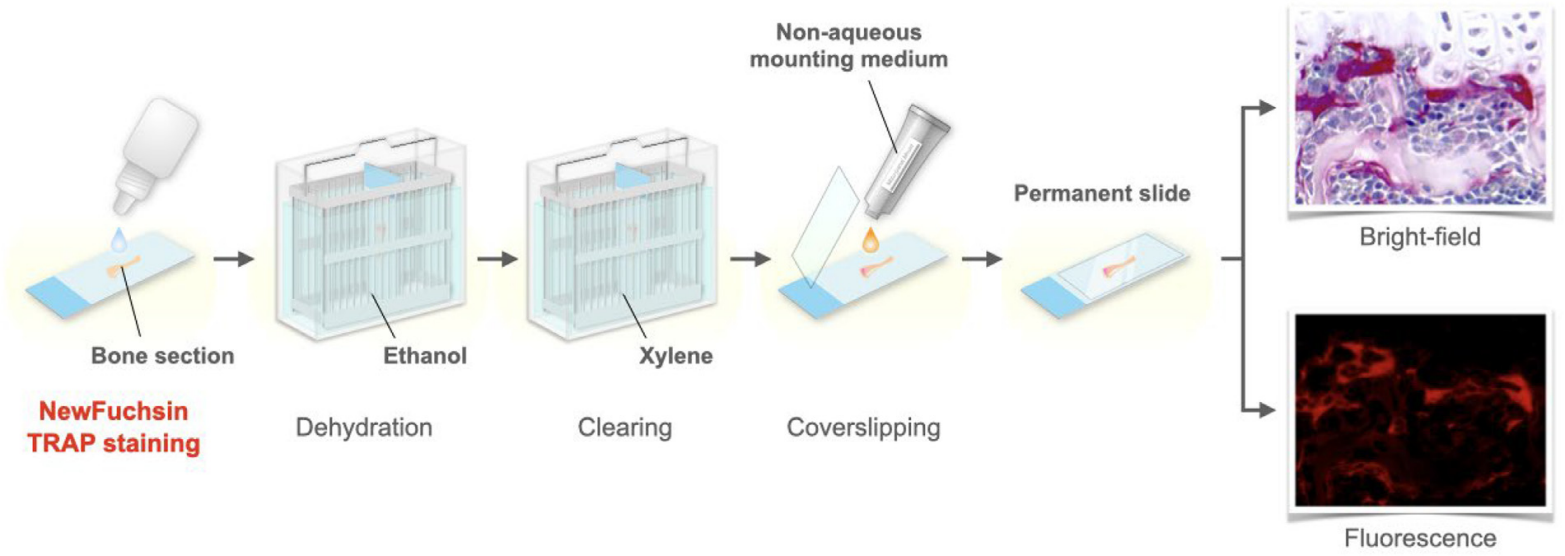
Preparation of permanent slides of bone sections with NewFuchsin TRAP staining. NewFuchsin TRAP staining of bone section samples allowed osteoclasts to be observed in bright-field and fluorescence, as well as for their long-term preservation.

Paraffin sections of decalcified mouse bone are prepared, stained with NewFuchsin TRAP, coverslipped in a non-aqueous mounting medium, and examined (Fig. 1).

#### Step 1–1: tissue collection and preparation

##### Materials

• 4% Paraformaldehyde solution (163-20145; FUJIFILM Wako Pure Chemical, Osaka, Japan)
• Ethylenediaminetetraacetic acid (EDTA, N001; DOJINDO Laboratories, Kumamoto, Japan)
• Ethanol (057-00456; FUJIFILM Wako Pure Chemical)
• Xylene (241-00091; FUJIFILM Wako Pure Chemical)
• Paraffin (Paraffin Wax II 60; Sakura Finetek, Tokyo, Japan)
• Microtome (REM-710; Yamato Kohki Industrial, Saitama, Japan)
• Slide warmer (PS-53; Sakura Finetek Japan)
• Coated glass slides (MAS-GP Type A; Matsunami Glass Industrial, Osaka, Japan)

The collected bones are fixed with paraformaldehyde and decalcified with EDTA, and paraffin sections are prepared.

1. Collect bones from mice sacrificed with overdose isoflurane and fix the samples overnight at 4 °C in a 4% paraformaldehyde solution.
2. Wash the bones with saline for 5 min. Repeat 3 times in total.
3. Incubate bones in 10% EDTA solution (pH 7.4) at 4 °C for 2 weeks; change the EDTA solution every few days.
4. Wash the decalcified bone with saline for 20 min. Repeat 3 times in total.
5. Dehydrate the bones with alcohol and xylene and embed in paraffin blocks.
6. Cut sections at a thickness of about 4–5 µm using a microtome.
7. Stretch the sections in a water bath at 40 °C for 1 h and transfer to slide glasses.
8. Dry the glass slides overnight on a slide warmer at 37 °C.

Tips: If sufficient TRAP activity is not obtained, warm the slides on a slide warmer at 37 °C for more than one week prior to deparaffinization. This procedure activates the enzymatic activity of TRAP.

#### Step 1–2: deparaffinization and hydration

##### Materials

• Ethanol
• Xylene

Deparaffinize and hydrophilize the slides by immersing them in the following solutions in sequence.

1. Immerse the slides in xylene for 10 min. Repeat 3 times in total.
2. Immerse the slides in 100% ethanol for 15 min. Repeat twice in total.
3. Incubate in 95% and 70% ethanol for 5 min each to hydrophilize the sections.
4. Wash the slides with tap water for 5 min. Repeat twice in total.

Tips: Insufficient deparaffinization leaves the tissue masked by paraffin and does not yield positive results. To avoid this, use fresh xylene and alcohol.

#### Step 1–3: preparation of stock solutions for NewFuchsin TRAP staining

##### Materials

• NewFuchsin (155,823; MP Biomedicals, Irvine, CA)
• Hydrogen chloride (083-02715; FUJIFILM Wako Pure Chemical)
• Sodium nitrite (191-02542; FUJIFILM Wako Pure Chemical)
• Naphthol AS-BI phosphate disodium salt hydrate (N2250; Sigma-Aldrich, St. Louis, MO)
• Sodium tartrate (190-03455; FUJIFILM Wako Pure Chemical)
• Sodium acetate (192-01075; FUJIFILM Wako Pure Chemical)

The following solutions are prepared as stock solutions.

• NewFuchsin stock solution (0.5% NewFuchsin in 2 M HCl)
• Sodium nitrite stock solution (0.4% sodium nitrite in distilled water)
• Naphthol AS-BI phosphate stock solution (5% naphthol AS-BI phosphate disodium salt in distilled water)
• TRAP buffer (50 mM sodium tartrate and 45 mM sodium acetate in distilled water)

The above stock solutions are stored at 4 °C.

#### Step 1–4: NewFuchsin TRAP staining

Diazotized NewFuchsin couples with naphthol AS-BI, produced by TRAP-mediated dephosphorylation, to form a red dye. The instructions include the preparation of approximately 1.0 mL of NewFuchsin TRAP staining working solution, which was sufficient to stain one slide.

1. Mix 10 µL of NewFuchsin stock solution and 10 µL of sodium nitrite stock solution in a micro test tube and incubate at room temperature for 1 min.
2. Add 1 mL of TRAP buffer.
3. Add 10 µL of naphthol AS-BI Phosphate stock solution and mix to make a working solution.
4. Gently drop the working solution onto the bone sections on the glass slides and incubate at room temperature for 10–20 min.
5. Rinse well with tap water.

Tips: If the coloration is weak, the reaction may be performed at 37 °C. A moisture chamber should be used to prevent drying of the sections.

#### Step 1–5: counterstaining (performed when nuclear staining is required)

##### Materials

• Mayer's hematoxylin solution (30011; Muto Pure Chemicals)

After immersion in Mayer's hematoxylin solution for 1–15 min, the samples are washed with running water at 55 °C for 15 min.

#### Step 1–6: dehydration and mounting

##### Materials

• Ethanol
• Xylene
• Coverslip (C024501; Matsunami Glass Industrial)
• Non-aqueous mounting medium (Marinol 750cps, 20092; Muto Pure Chemicals)

After dehydration, mount with non-aqueous mounting medium.

1. Immerse the slides in 70% ethanol for 3 min.
2. Immerse the slides in 100% ethanol for 3 min. Repeat 3 times in total.
3. Immerse the slides in xylene for 3 min. Repeat twice in total.
4. Add non-aqueous mounting medium to slides and top with coverslips.

Tips: Use coverslips pre-dipped in xylene to prevent air bubbles from remaining during mounting.

#### Step 1–7: observation

Microscopy is used to examine specimens. When observing the fluorescence, use an excitation filter for rhodamine.

### Whole-mount NewFuchsin TRAP staining of bone tissue

**Fig. 2:**
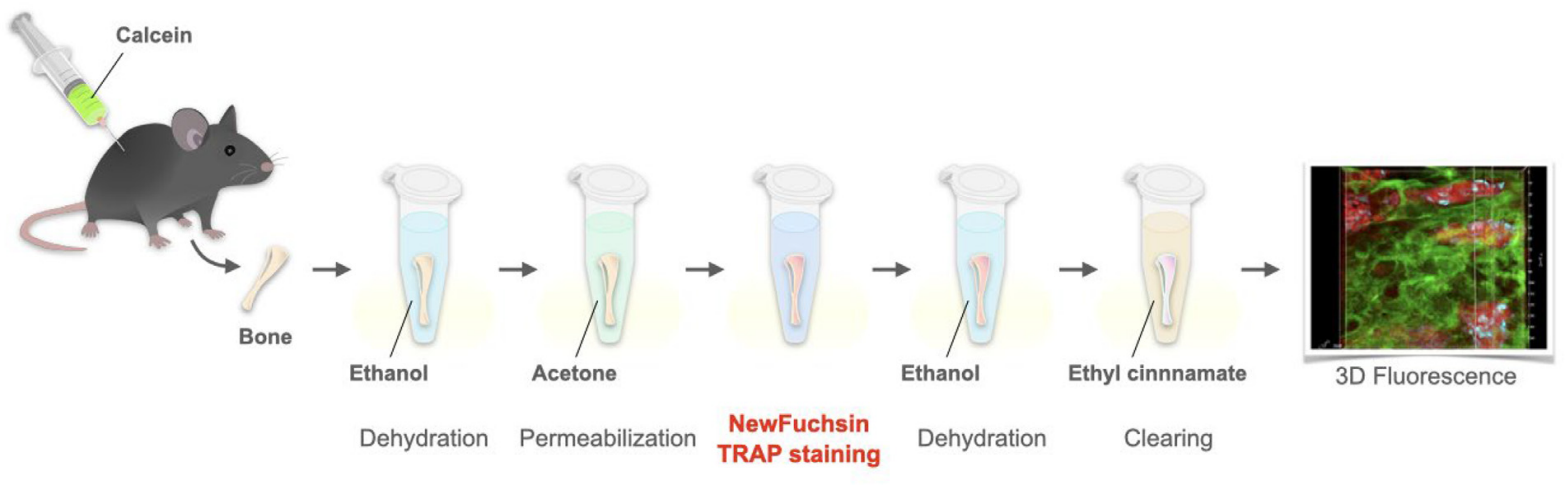
Scheme of NewFuchsin TRAP staining and tissue clearing of calcein-labeled whole bone. Ethyl cinnamate clearing of NewFuchsin TRAP-stained bone with calcein labeling allows the visualization of osteoclast localization and osteogenic sites. This process is performed in a 1.5-mL polypropylene tube.

To visualize osteoclasts in whole bone, NewFuchsin TRAP-stained bone is observed using fluorescence microscopy after tissue clearance with ethyl cinnamate [4]. This method can be used in combination with fluorescent proteins and fluorescent-labeled antibodies. This section describes the combination of calcein labeling used to detect the site of bone formation (Fig. 2).

#### Step 2–1: calcein labeling and tissue collection

##### Materials

• Calcein (C001; DOJINDO Laboratories)
• 4% Paraformaldehyde solution

1.6 mg/mL Calcein solution is prepared as follows.

1. Dissolve calcein in 0.1 M KOH and adjust to pH 7.0 with NaOH to make a 10 mg/mL stock solution.
2. Dilute with saline to 1.6 mg/mL before use.
3. Excise fibulae from calcein-treated mice and fix them with paraformaldehyde.
4. Administer 1.6 mg/mL calcein solution (10 µL/g of body weight) intraperitoneally to 3-week-old male mice.
5. Two days later, sacrifice the mice and remove the fibula with the tibia attached.
6. Fix bone in 4% paraformaldehyde solution overnight at 4 °C.
7. Use a razor blade to create an appropriate split surface to improve the penetration of solutions.

Subsequent steps use 1.5-mL polypropylene microcentrifuge tubes.

#### Step 2–2: dehydration and NewFuchsin TRAP staining

##### Materials

• Ethanol
• Acetone (016–00346; FUJIFILM Wako Pure Chemical)
• NewFuchsin TRAP staining solution diluted 100× in TRAP buffer (1/100 substrate concentration of the NewFuchsin TRAP staining working solution described in Step 1–4)

Tissues are pre-dehydrated with ethanol for rapid permeation of TRAP staining solution.

1. Immerse the bone in 1 mL of 70% ethanol for 60 min.
2. Immerse the bone in 1 mL of 80% ethanol for 60 min.
3. Immerse the bone in 1 mL of 90% ethanol for 60 min.
4. Immerse the bone in 1 mL of 95% ethanol for 60 min.
5. Immerse the bone in 1 mL of 100% ethanol for 60 min. Repeat 3 times in total.
6. Immerse the bone in 1 mL of ethanol/acetone (1:1 by volume) for 6 h.
7. Immerse the bone in 1 mL of acetone for 12 h.
8. Immerse the bone in 1 mL of 100% ethanol for 60 min. Repeat twice in total.
9. Immerse the bone in 1 mL of 70% ethanol for 60 min. Repeat twice in total.
10. Immerse the bone in 1 mL of ice-cold 100x diluted NewFuchsin TRAP staining solution for 10 min.
11. Change temperature to 37 °C and incubate for 30 min.
12. Wash the bone with ice-cold water for 60 min. Repeat this step twice for a total of three washes.

#### Step 2–3: counterstaining (performed when nuclear staining is required)

##### Materials

• Ethanol
• 4′,6-diamidino-2-phenylindole (DAPI, D9542; Sigma-Aldrich)

After TRAP staining, immerse the bone in 70% ethanol for 60 min, then immerse in DAPI solution (3 µg/mL DAPI in 70% ethanol) for 2 days and wash with 70% ethanol for 60 min.

#### Step 2–4: dehydration and tissue clearing

##### Materials

• Ethanol
• Ethyl cinnamate (112372; Sigma-Aldrich)

Thoroughly dehydrate over time to ensure successful clearing with ethyl cinnamate.

1. Immerse the bone in 1 mL of 70% ethanol for 2 h.
2. Immerse the bone in 1 mL of 80% ethanol for 2 h.
3. Immerse the bone in 1 mL of 90% ethanol for 2 h.
4. Immerse the bone in 1 mL of 95% ethanol for 2 h.
5. Immerse the bone in 1 mL of 100% ethanol for 2 h. Repeat 3 times in total.
6. Immerse the bone in 1 mL of ethyl cinnamate for 1 h. Repeat twice in total.
7. Immerse the bone in 1 mL of ethyl cinnamate for 24 h. Repeat twice in total.

Tips: Some of the red dye leaches out during the initial ethyl cinnamate immersion; therefore, the solution should be changed frequently to reduce background staining.

#### Step 2–5: observation

Cleared bone is observed using confocal laser scanning microscopy equipped with fluorescence detector.

## Method validation

**Fig. 3:**
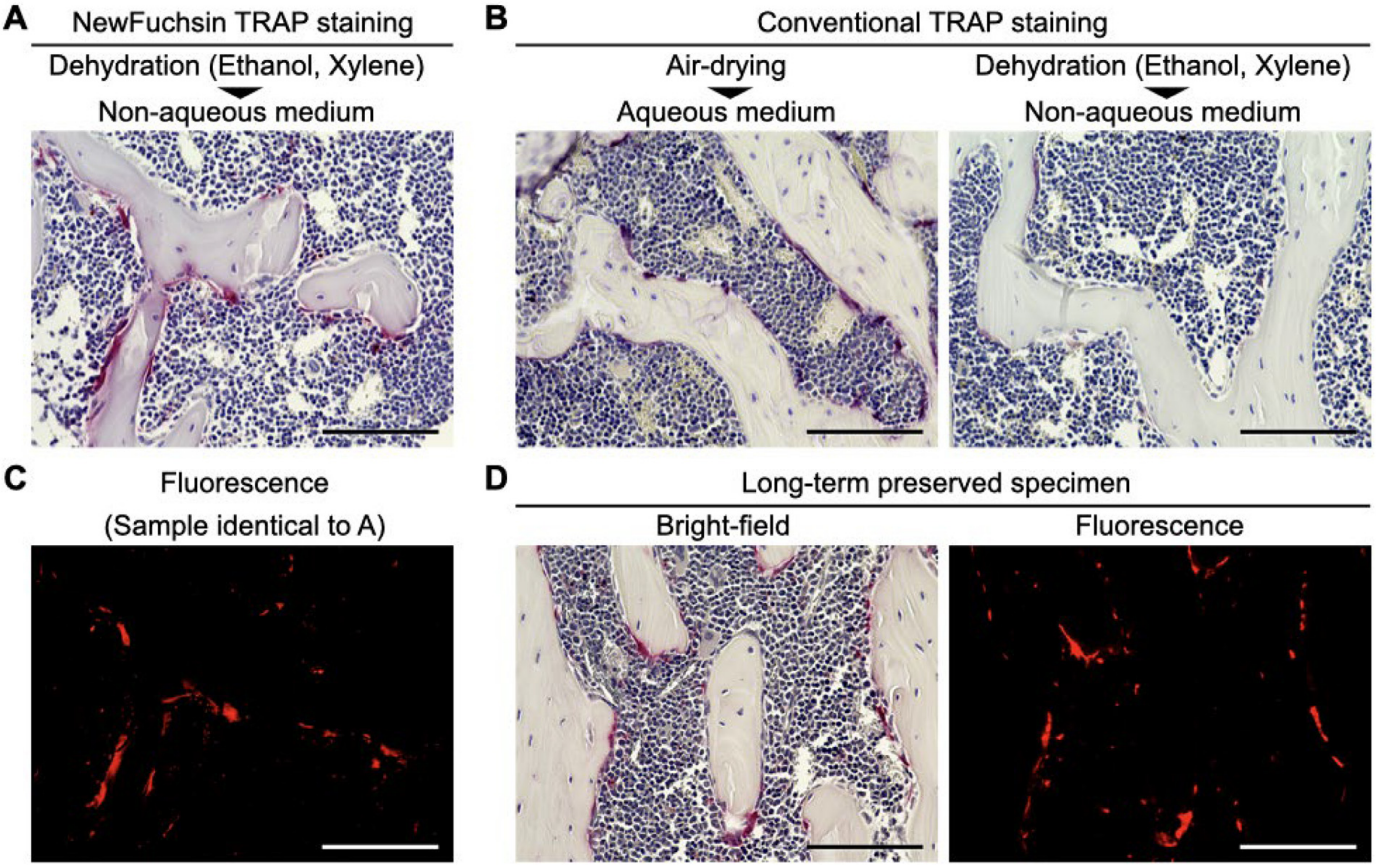
Organic solvent-resistant TRAP staining provides both bright-field and fluorescence viewing of specimens that withstands long-term preservation. (A–D) NewFuchsin TRAP staining of trabecular bone in proximal tibial metaphysis of 12-week-old male mice. (A) NewFuchsin TRAP staining followed by ethanol and xylene immersion and coverslip with non-aqueous mounting medium. (B) Conventional TRAP staining followed by air drying and aqueous mounting medium (left) and ethanol and xylene immersion and coverslip with non-aqueous mounting medium (right); scale, 100 µm. (C) Fluorescence observation of NewFuchsin TRAP-stained bone section immersed in ethanol and xylene and coverslipped with non-aqueous mounting medium (sample identical to A); scale, 100 µm. (D) Bright-field and fluorescence observations of a specimen stored at room temperature for 24 months after NewFuchsin TRAP staining; scale, 100 µm.

In this report, we present a method for the preparation of long-term stable TRAP-stained bone specimens that can be observed using both fluorescence and bright fields. To validate this method, NewFuchsin TRAP staining was performed on sections of decalcified femora extracted from 12-week-old C57BL/6J mice according to the method shown above. For comparison, conventional TRAP staining was performed using fast garnet GBC chromogen and naphthol AS-BI phosphate. NewFuchsin TRAP staining provided a clear image of the stained osteoclasts on the bone surface, even after immersion in ethanol and xylene (Fig. 3A). In contrast, conventional TRAP-stained specimens showed stained osteoclasts when coverslipped with aqueous mounting medium after air-drying, but the tissue showed a non-specific yellowish staining (Fig. 3B, left). Furthermore, the red staining of osteoclasts almost disappeared when the coverslips were covered with a non-aqueous mounting medium after immersion in ethanol and xylene for dehydration (Fig. 3B, right). Specimens subjected to NewFuchsin TRAP staining were observed for fluorescence (Fig. 3C). Specimens with NewFuchsin TRAP staining withstood storage for more than one year. Specimens stored at room temperature for 24 months after staining showed hematoxylin discoloration, but stained osteoclasts were clearly visible in both bright-field and fluorescence (Fig. 3D).

**Fig. 4:**
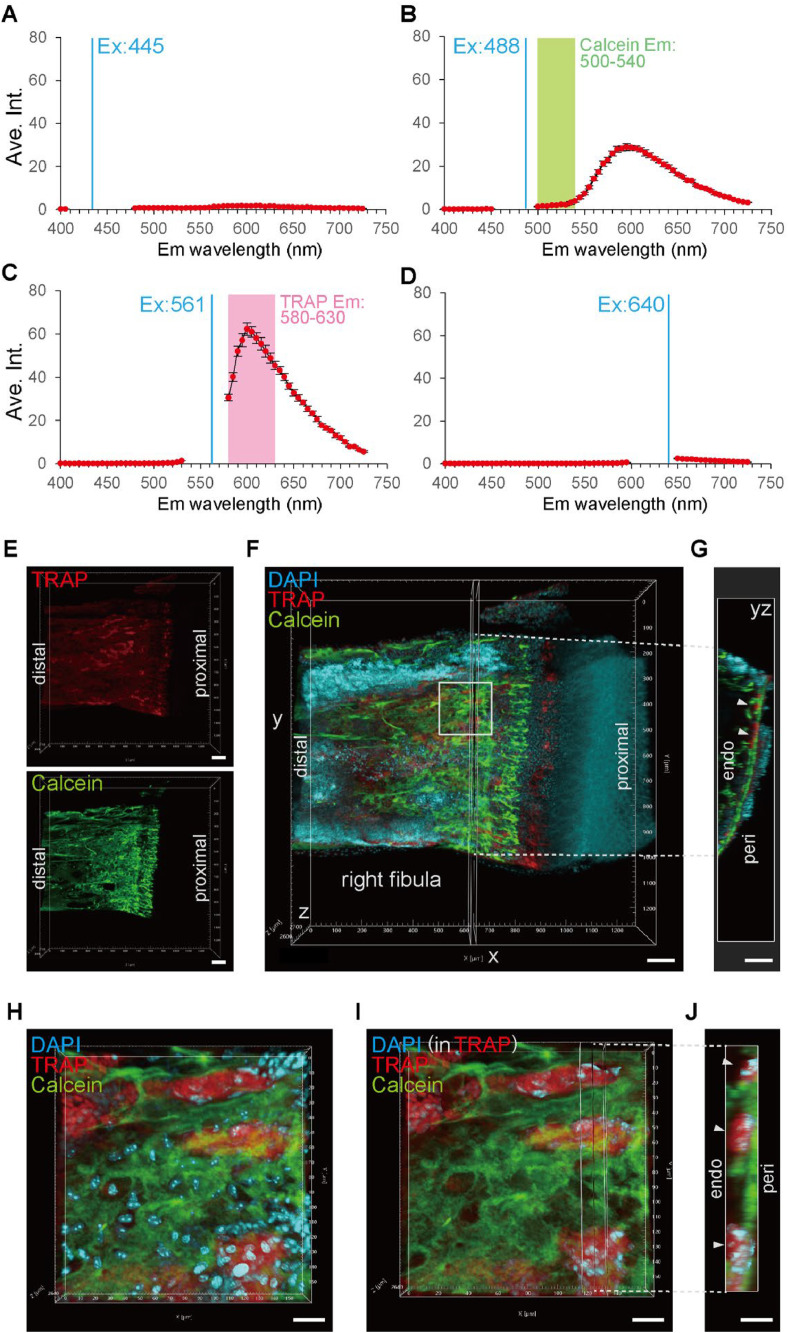
Fluorescence imaging of NewFuchsin TRAP staining. (A–D) Average emission (Em) spectrums of NewFuchsin TRAP staining by excitation (Ex) with 445 nm (A), 488 nm (B), 561 nm (C) or 640 nm (D) laser. Mean ± standard error, *n* = 5. The detection range for calcein imaging (E–J) was shown in (B). The detection range for TRAP imaging (E–J) was shown in (C). (E,F) The 205 µm-thick 3D-reconstructed images (*Z* = 42, interval 5 µm) of NewFuchsin TRAP staining with calcein labeling in the fibula of a 3-week-old male mouse under confocal laser scanning microscopy; scale, 100 µm. (G) The maximum intensity projection image of the 30 µm-thick digital *yz*-slice indicated in (F); scale, 100 µm. (H–J) Magnified views of the boxed area in (F); scale, 20 µm. (H) The 20 µm-thick 3D-reconstructed images (*Z* = 23, interval 0.91 µm) are shown. (I) The DAPI signal outside of TRAP was obscured. (J) Maximum intensity projection image of the 16 µm-thick digital *yz*-slice indicated in (I); arrowheads, osteoclasts.

Taking advantage of the organic solvent resistance of the NewFuchsin TRAP stain, we tested a tissue clearing technique using ethyl cinnamate for 3D analysis. Samples cleared with ethyl cinnamate were observed under an FV4000 confocal laser scanning microscope (Evident, Tokyo, Japan). In order to achieve simultaneous imaging of NewFuchsin TRAP stain and calcein, a lambda scanning was conducted to measure the emission spectrum of NewFuchsin TRAP stain (Fig. 4A–D). The 445-nm laser exhibited minimal emission (Fig. 4A). The sample was partially excited at 488 nm, but the fluorescence spectrum did not overlap with the calcein detection range (Fig. 4B). NewFuchsin TRAP fluorescence was excited using a 561-nm laser and observed at 580–630 nm for fluorescence imaging (Fig. 4C). The 640-nm laser exhibited minimal emission (Fig. 4D). By combining the NewFuchsin TRAP staining, tissue clearing technique, and calcium labeling of bone formation sites, we were able to simultaneously visualize the bone formation and resorption sites (Fig. 4E–G). The visualization of multinucleated osteoclasts in non-decalcified 3D bone tissue was successfully achieved by NewFuchsin TRAP staining (Fig. 4H–J).

## Limitations

TRAP is particularly abundant in osteoclasts but is also present in non-osteoclasts [5]. Compared to paraffin-embedded bone sections, non-osteoclasts appear to stain slightly more in whole-mount bone. Whole-bone TRAP staining is possible for calcein-labeled bone; however, prolonged exposure to acidic TRAP solutions causes decalcification. To avoid the loss of the calcein label due to decalcification, it is helpful to adjust the permeation and reaction time according to size of the sample. The reaction products of NewFuchsin and naphthol AS-BI were well excited by the 561-nm laser, but were also slightly excited at 488 nm. Therefore, the use of a long-pass filter for calcein imaging is inappropriate for calcein-labeled bones.

## Ethics statements

C57BL/6J male mice were purchased from the Sankyo Labo Service Corporation (Tokyo, Japan). All experiments were reviewed and approved by the Animal Care and Use Committee of the Tokyo Dental College (No. 300402). This study was conducted in accordance with the ARRIVE guidelines and the National Institutes of Health Guide for the Care and Use of Laboratory Animals (NIH Publication No. 8023; revised 1978).

## CRediT authorship contribution statement

**Takashi Nakamura:** Conceptualization, Methodology, Validation, Writing – original draft. **Katsuhiro Kawaai:** Methodology, Validation, Writing – review & editing. **Yukiko Kuroda:** Methodology, Validation, Writing – review & editing. **Koichi Matsuo:** Conceptualization, Methodology, Validation, Supervision, Writing – review & editing.

## Declaration of competing interest

The authors declare that they have no known competing financial interests or personal relationships that could have appeared to influence the work reported in this paper.
